# Esmolol acutely alters oxygen supply‐demand balance in exercising muscles of healthy humans

**DOI:** 10.14814/phy2.13673

**Published:** 2018-04-17

**Authors:** David N. Proctor, J. Carter Luck, Stephan R. Maman, Urs A. Leuenberger, Matthew D. Muller

**Affiliations:** ^1^ Noll Laboratory Department of Kinesiology The Pennsylvania State University University Park Pennsylvania; ^2^ Penn State Heart and Vascular Institute Penn State University College of Medicine Hershey Pennsylvania; ^3^ Master of Science in Anesthesia Program Case Western Reserve University School of Medicine Cleveland Ohio

**Keywords:** Beta‐adrenergic blockade, blood pressure, heart rate, near‐infrared spectroscopy, oxygen uptake

## Abstract

Beta‐adrenoreceptor antagonists (*β* blockers) reduce systemic O_2_ delivery and blood pressure (BP) during exercise, but the subsequent effects on O_2_ extraction within the active limb muscles are unknown. In this study, we examined the effects of the fast‐acting, *β*_1_ selective blocker esmolol on systemic hemodynamics and leg muscle O_2_ saturation (near infrared spectroscopy, NIRS) during submaximal leg ergometry. Our main hypothesis was that esmolol would augment exercise‐induced reductions in leg muscle O_2_ saturation. Eight healthy adults (6 men, 2 women; 23–67 year) performed light and moderate intensity bouts of recumbent leg cycling before (PRE), during (*β*_1_‐blocked), and 45 min following (POST) intravenous infusion of esmolol. Oxygen uptake, heart rate (HR), BP, and O_2_ saturation (SmO_2_) of the vastus lateralis (VL) and medial gastrocnemius (MG) muscles were measured continuously. Esmolol attenuated the increases in HR and systolic BP during light (−12 ± 9 bpm and −26 ± 12 mmHg vs. PRE) and moderate intensity (−20 ± 10 bpm and −40 ± 18 mmHg vs. PRE) cycling (all *P* < 0.01). Exercise‐induced reductions in SmO_2_ occurred to a greater extent during the *β*_1_‐blockade trial in both the VL (*P* = 0.001 vs. PRE) and MG muscles (*P* = 0.022 vs. PRE). HR, SBP and SmO_2_ were restored during POST (all *P* < 0.01 vs. *β*_1_‐blocked). In conclusion, esmolol rapidly and reversibly increases O_2_ extraction within exercising muscles of healthy humans.

## Introduction

Beta‐adrenoreceptor antagonist drugs (*β* blockers) are the standard of care for patients with cardiac diseases such as previous myocardial infarction, stable angina, and heart failure (Freemantle et al. 1999; Frishman 2013; de Shu et al. 2012). Beta blockers reduce myocardial oxygen demand by reducing HR (Wellstein et al. 1987) and contractility (Weidemann et al. 2002). However, these cardiac effects can also reduce the exercise tolerance in people taking these medications due to limitations in systemic O_2_ delivery (via an impaired ability to raise heart rate and cardiac output) and active muscle blood flow (via a reduction in local perfusion pressure and/or augmented sympathetic vasoconstriction) (Hughson and Kowalchuk 1991; Pawelczyk et al. 1992; Tesch 1985). These hemodynamic effects are particularly evident during whole body exercise when compensatory increases in stroke volume are insufficient to preserve cardiac output and when sympathetic outflow increases to active skeletal muscles (Gullestad et al. 1996; Hughson and Kowalchuk 1991; MacFarlane et al. 1983; Tesch 1985). For people taking *β* blockers and performing activities of daily living (e.g. walking, climbing stairs, yard/house work), further widening of the arteriovenous O_2_ difference becomes an important compensatory mechanism for meeting O_2_ demand in peripheral tissues (Hughson and Kowalchuk 1991).

Compensatory widening of the systemic arterial‐venous O_2_ difference in response to acute *β* blockade in exercising humans has been recognized since the 1960s (Epstein et al. 1965; Hamer and Sowton 1965). This response is thought to reflect, in large part, augmented extraction of O_2_ within the active muscle groups secondary to a reduction in muscle blood flow and O_2_ delivery (Hughson and Kowalchuk 1991; Tesch 1985). To the best of our knowledge only two studies have measured active limb venous O_2_ content/O_2_ extraction during large muscle mass exercise in *β*‐blocked humans (Pawelczyk et al. 1992; Pirnay et al. 1972); both studies reported lower venous O_2_ content in the femoral vein of healthy adults following systemic *β*_1_ blockade, during submaximal cycling (Pawelczyk et al. 1992) and maximal treadmill (Pirnay et al. 1972) exercise. Pawelczyk and colleagues further showed that *β*_1_ blockade reduced whole leg blood flow and O_2_ delivery and that the augmented O_2_ extraction across the exercising legs served to maintain leg O_2_ uptake. These findings suggest that *β*_1_ blockade does indeed affect O_2_ delivery and O_2_ extraction (in a reciprocal manner) within the active limbs of *β*‐blocked humans. While direct, these whole limb measures of O_2_ extraction/venous O_2_ content do not indicate specifically where these *β*_1_‐induced alterations in limb O_2_ supply‐demand are taking place (i.e., active vs. less active tissues), nor do they provide insight into the dynamic nature (onset, maintenance, reversibility, exercise intensity dependence) of these responses. Insight into these questions would advance our understanding of the local compensatory effects of these widely used drugs.

In this study, we addressed these gaps in knowledge using esmolol, a fast‐acting *β*_1_ selective blocker, in combination with the high temporal resolution of near infrared spectroscopy (NIRS). Specifically, we investigated the effects of esmolol on deoxygenation in two leg muscles (vastus lateralis, VL; medial gastrocnemius, MG) and systemic O_2_ uptake and hemodynamics during light and moderately intense cycle exercise in healthy adults. Esmolol hydrochloride (Brevibloc, Baxter Healthcare) is commonly used in clinical practice, with an elimination half‐life of <10 min due to metabolism by red blood cell esterases (Harless et al. 2015; Sum et al. 1983). We recently established that infusion of ~160 mg of esmolol (range 110–200 mg in the 5 min before exercise) acutely and selectively blocks *β*_1_ receptors (Muller et al. 2017). The rapid half‐life of esmolol permitted us to assess SmO_2_ in the presence and subsequent absence of esmolol in a single visit, while keeping the NIRS probes in a constant location. We intentionally studied acute responses in healthy adults who were normally active to avoid the potentially confounding effects of chronic disease and *β*‐blocker use (Eynon et al. 2008), as well as aerobic exercise training (Proctor et al. 2001), on exercising muscle O_2_ extraction. In this way the intrinsic responses of exercising human muscle to an acute reduction in systemic O_2_ supply could be elucidated. We hypothesized that esmolol would attenuate exercise‐induced increases in HR and BP and evoke a rapid augmentation of O_2_ extraction in the active muscles of healthy humans, and that these effects would be quickly reversed due to the short half‐life of this drug.

## Methods

### Subjects and study design

Subjects in this study were part of a larger investigation examining the HR lowering effects of esmolol versus propranolol (Muller et al. 2017) and included healthy men and women who had undergone clinical screening tests (medical history, physical exam, and blood draw) and a graded treadmill test to maximal exertion prior to participating in the present protocol. Subjects who screened into this study ranged from 23 to 67 years of age, were nonsmokers, nonobese, and not taking any medications. Exclusion criteria included: pregnant and nursing women, individuals with a resting HR <45 beats/min, fat free mass (FFM) >100 kg, a history of cardiovascular, pulmonary, renal, or endocrine disease. Physical characteristics are provided in Table 1.

**Table 1:** Subject characteristics

Variable	
Sample, male/female	6/2
Age, years	40 (23–68)
Height, m	1.77 (1.55–1.93)
Weight, kg	79.9 (58.2–101.9)
Body mass index	25.1 (22.4–30.0)
Body fat, %	19.5 (10.5–28.2)
Fat‐free mass, kg	64.1 (45.0–81.3)
Peak VO_2_, mL/kg per min	43.2 (27.5–55.6)
Systolic blood pressure, mmHg	113 (104–130)
Diastolic blood pressure, mmHg	69 (61–83)
Mean arterial pressure, mmHg	83 (75–91)
Heart rate, beats/min	60 (44–76)
Hemoglobin, g/dl	13.9 (12.7–15.6)
Hematocrit, %	40.1 (37.5–44.3)
Adipose tissue thickness, mm	
Vastus lateralis	6.15 (3.54–9.23)
Medial gastrocnemius	4.15 (2.99–6.29)

Values are means with min‐max values in parentheses.

These laboratory experiments used a repeated‐measures design where each participant served as his/her own control. Participants performed exercise bouts before (PRE), during (*β*_1_‐blocked), and 45 min following (POST) intravenous infusion of esmolol hydrochloride (Brevibloc, Baxter Healthcare). This “Off‐On‐Off” paradigm was facilitated by the rapid onset (<2 min) and short half‐life (<10 min) of esmolol. All drug doses were calculated using FFM determined by dual‐energy X‐ray absorptiometry (DEXA). The two women in this study were both premenopausal.

All protocols were approved by the Institutional Review Board of Penn State University College of Medicine, in accordance with the Declaration of Helsinki. Each subject voluntarily gave their written, informed consent prior to participation in this study.

### Protocol and methods

**Figure 1:**
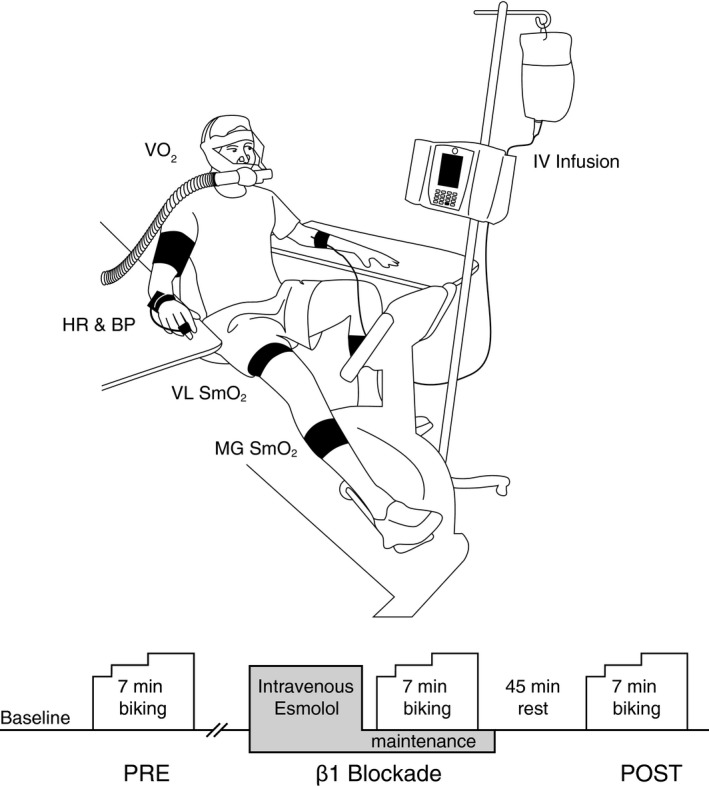
Diagram of experimental setup and timeline. VO_2_, oxygen uptake; HR, heart rate; BP, blood pressure; VL, vastus lateralis muscle; MG, medial gastrocnemius muscle; SmO_2_, skeletal muscle oxygenation.

These experiments were conducted in a thermoneutral (20–22°C) Clinical Research Center laboratory after subjects fasted ≥4 h. Subjects were instructed to refrain from exercise, caffeine, and alcohol for a minimum of 24 h before the experimental visit. BP was measured in triplicate using an automated oscillometric cuff (vital signs monitor SureSigns VS3, Philips) following a ten to fifteen minute rest period in the supine posture. Subjects were transitioned to the semi‐recumbent position on a cycle ergometer (Nautilus NR 2000; Fig. 1) and were instrumented with a three‐lead electrocardiogram (Cardiocap/5, GE Healthcare) and noninvasive brachial BP measured by an automated auscultatory cuff (SunTech Tango). A finger BP cuff (Finometer, Finapres Medical Systems) was attached for beat‐to‐beat analysis. A 22 g intravenous catheter was placed in the deep antecubital fossa of each subject's left arm. Two arm tables were raised to heart level and supported both arms to reduce motion artifact and optimize comfort. Pulmonary gas exchange was measured using an automated metabolic gas analyzer (ParvoMedics). Oxygen pulse, an index of stroke volume, was calculated as systemic O_2_ consumption expressed in mL/min divided by HR expressed in beats/min (Crisafulli et al. 2007). Two continuous wave NIRS devices (Moxy, Fortiori Design LLC) were attached to the right calf and right thigh at the region of the medial gastrocnemius and vastus lateralis muscles, respectively. The NIRS provides a measure of microvascular muscle oxygen saturation (SmO_2_) where SmO_2_ is the ratio of oxygenated hemoglobin to total hemoglobin expressed as a percentage. The NIRS technique has been extensively reviewed (Ferrari et al. 2011; Grassi and Quaresima 2016; Hamaoka et al. 2011; Jones et al. 2016) and previously described by our lab (Luck et al. 2017). Briefly, the NIRS device used in this study utilizes a four layer (epidermis, dermis, adipose, and muscle) Monte Carlo simulated model to trace the propagation of photons through a tissue medium. Wavelengths of 680, 720, 760, and 800 nm with a source‐detector separation of 12.5 and 25 mm were used. The NIRS signal penetration depth (~12.5 mm) was approximately half of the source‐detector separation distance. Penetrating light absorbed primarily by oxygenated hemoglobin, deoxygenated hemoglobin, and myoglobin was detected using the NIRS optical absorbance spectrum. The absorption spectrum of Mb is reported to overlap with Hb and therefore both molecules contributed to changes in the NIRS signal (Grassi and Quaresima 2016). Adipose tissue thicknesses (ATT) were obtained using ultrasound imaging (iE33, Philips) at each NIRS probe site to ensure proper signal penetration depth. In 2D mode, an average of three manually selected distances were measured from the epidermis to the superficial aponeurosis using a L11‐3 linear array transducer.

Following a three minute baseline and 30‐sec passive warm‐up at 30 rpm (to overcome initial inertia on the belt drive mechanism), participants performed 3 min of light and three and a half minutes of moderately intense bouts of steady‐state recumbent leg cycling before (PRE), during (*β*_1_‐blocked), and 45‐min following (POST) intravenous infusion of esmolol. Pedaling cadence was 60 rpm and was monitored by the participant and laboratory personnel via digital display. Light and moderately intense bouts of exercise were determined by a target HR of 110 beats/min and 150 beats/min, respectively. Exercise and infusion protocols have been previously described (Muller et al. 2017) and are outlined in Figure 1. Based on data obtained in previous studies and clinical guidelines (Muller et al. 2017), we chose to administer esmolol hydrochloride (2500 mg in 250 mL) intravenously as 0.5 mg/kg FFM/min for 3 min followed by 0.25 mg/kg FFM/min maintenance dose.

### Data collection and statistical analysis

HR and BP were collected continuously at 200 Hz and analyzed offline (Powerlab 16SP, ADInstruments). NIRS SmO_2_ was measured continuously at 2 Hz and transmitted wirelessly via ANT+ to a laptop computer for offline analysis (PeriPedal v2.4.8). Pulmonary gas exchange was measured and analyzed offline (ParvoMedics).

Statistics were performed using IBM SPSS 24.0. A 3 drug treatment (PRE, *β*‐blocked, POST) × 3 intensity (baseline, light intensity, moderate intensity) repeated measures ANOVA was conducted on the raw physiological measures. When a significant drug x intensity interaction was observed, paired *t*‐tests were conducted for the moderate intensity time point only. To determine effect sizes, Cohen's *d* values were calculated as *t*‐statistic/square root of the sample size. Cohen's d values > 0.8 were considered to be “strong” effect sizes. Data are shown as mean ± standard deviation unless otherwise noted. *P* < 0.05 was considered statistically significant.

## Results

Eight subjects participated in this study. Physical characteristics and peak cycle exercise responses are provided in Table 1 and reflect the fact that these participants were healthy with average levels of cardiorespiratory fitness.

**Figure 2:**
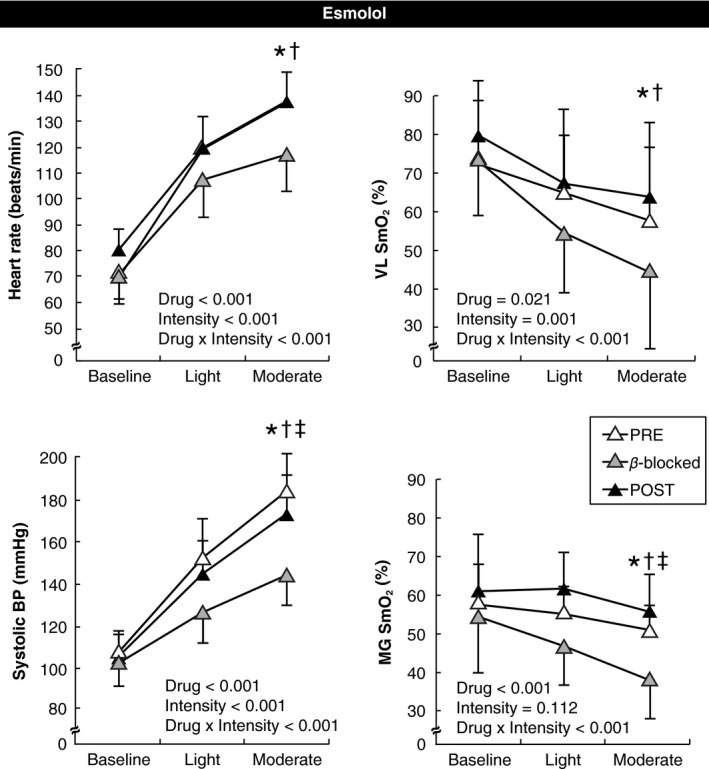
Heart rate, systolic BP, and muscle oxygen saturation (SmO_2_) responses at baseline rest (seated on bike), and during light and moderate intensity cycling during the esmolol study visit. PRE, responses measured before esmolol was infused. *β*‐blocked, responses measured during esmolol maintenance infusion. POST, responses measured ~45 min after esmolol infusion was discontinued. ^*^ *P* < 0.05 comparing PRE to Beta‐ blocked; ^†^ *P* < 0.05 comparing POST to Beta‐blocked; ^‡^ *P* < 0.05 comparing PRE to POST.

**Figure 3:**
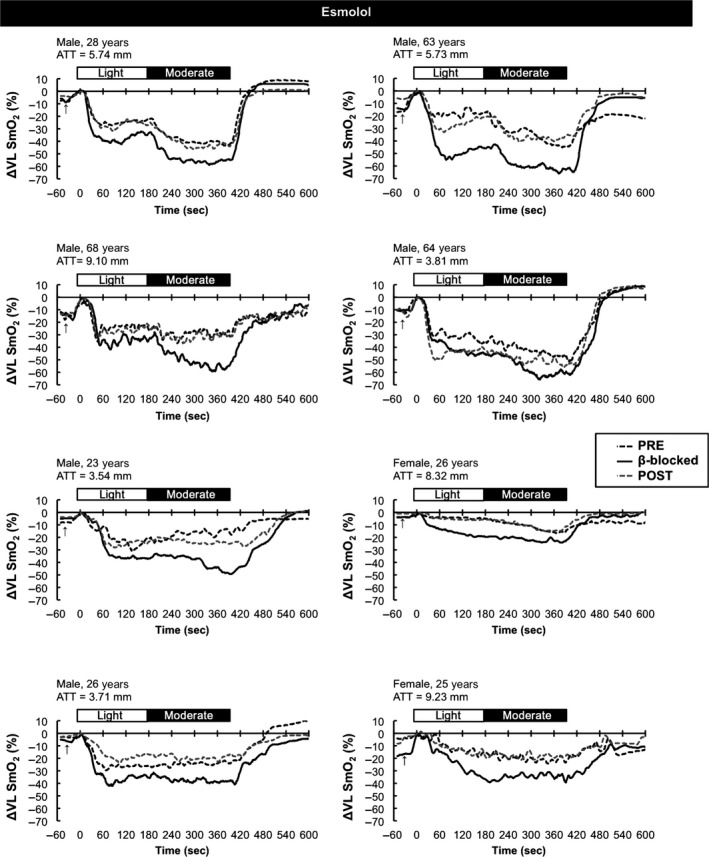
Individual oxygen saturation response patterns in the vastus lateralis (VL) muscle from the esmolol study visit. PRE (dashed black line), *β*‐blocked (solid black line), and POST (dashed gray line) trial values were normalized to the % saturation obtained at the end of the 30 sec assisted pedaling phase for each trial (zeroed to the horizontal line). ATT, adipose tissue thickness over the VL muscle.

As expected, the steady state respiratory and RPE responses to exercise were similar before, during, and after *β*1 blockade with esmolol (Table 2, all *P* > 0.5). However, compared to preblockade conditions, O_2_ pulse was greater with *β*‐1 blockade (i.e., suggestive of greater stroke volume) at both light (*P* = 0.008, Cohen's d = 1.60) and moderate (*P* = 0.009, Cohen's d = 1.10) workloads. As noted in Figure 2, the HR response to exercise was attenuated by esmolol (*P* < 0.001 compared to the Pretrial) and this HR response was restored 45 min after ending the esmolol infusion (*P* < 0.001 compared to beta‐blocked trial). In a similar way, the systolic BP response was attenuated by esmolol (*P* < 0.001 compared to the Pretrial) and was restored 45 min after ending the esmolol infusion (*P* = 0.001 compared to beta‐blocked trial). As noted in Figure 2, NIRS responses in the VL and MG muscle were lower (i.e., more oxygen extraction) during beta‐blockade with esmolol compared to preblockade (*P* = 0.001 for the VL and *P* = 0.022 for the MG, Cohen's d = 0.76 and 1.86, respectively). These NIRS responses were restored 45 min after ending the esmolol infusion in both the VL muscle (*P* = 0.001, Cohen's d = 2.22) and MG muscle (*P* = 0.001, Cohen's d = 3.60). NIRS responses for each subject are shown in Figure 3.

**Table 2:** Effect of esmolol on systemic responses to light and moderate intensity leg cycling

	PRE	*β*1 blockade	POST
VO_2_ (L/min)			
Light	1.35 ± 0.13	1.37 ± 0.09	1.42 ± 0.10
Moderate	1.75 ± 0.13	1.73 ± 0.18	1.76 ± 0.14
Oxygen pulse (mL O_2_/beat)			
Light	11.5 ± 1.4	13.1 ± 2.2^a^	12.1 ± 1.8
Moderate	12.9 ± 1.7	15.0 ± 2.8^a^	12.9 ± 1.8
RER			
Light	0.94 ± 0.10	0.85 ± 0.09	0.84 ± 0.08
Moderate	1.03 ± 0.04	1.00 ± 0.06	0.99 ± 0.05
RPE			
Light	12 (10–13)	12 (10–14)	10 (8–13)
Moderate	14 (13–18)	16 (14–19)	16 (13–18)

VO_2_, oxygen uptake; RER, respiratory exchange ratio; RPE, rating of perceived exertion. Data are mean ± SD except for RPE which is median (min‐max).

a Indicates *P* < 0.05 compared to Pretrial.

## Discussion

This study examined the effects of the short‐acting *β*_1_‐blocker esmolol on NIRS‐based estimates of active muscle O_2_ saturation during large muscle mass exercise. Consistent with our hypothesis, esmolol infusion at a dose sufficient to reduce exercise HR ~12–20 beats/min (10–15%) produced an O_2_ supply‐demand mismatch that evoked greater leg exercise‐induced reductions in SmO_2_ within the active muscles of healthy adults. The augmented drop in leg SmO_2_ (1) occurred rapidly, (2) was observed during both work intensities in most of these subjects, (3) was observed in both muscles examined, and (4) was quickly reversible. These findings provide strong evidence of the dynamic coupling that exists between systemic O_2_ delivery and active muscle O_2_ extraction when systemic demand for blood flow is high. This study also demonstrates the utility of these combined methods for investigating acute adjustments in O_2_ delivery and peripheral O_2_ extraction in exercising humans.

### Effects of esmolol on exercising muscle O_2_ saturation

A widening of the systemic arteriovenous O_2_ difference has long been recognized as a compensatory adjustment during exercise in *β*–blocked humans (Gullestad et al. 1996; Hughson and Kowalchuk 1991; MacFarlane et al. 1983; Tesch 1985). Pawelczyk et al. (1992) observed a lower femoral venous O_2_ content during submaximal cycling in healthy young adults following metoprolol infusion, supporting the idea that the legs are likely contributing to this augmented systemic O_2_ extraction. This study is the first to directly assess the effects of systemic *β*‐blockade on O_2_ extraction within individual exercising human muscles. Our use of NIRS in combination with esmolol revealed an augmented drop in leg SmO_2_ that was remarkably sensitive to changes in muscle oxygen demand (i.e., exercise intensity and restoration after metabolism of the drug). The rapid and reversible nature of this augmented deoxygenation response, its consistency across all 8 subjects studied (Fig. 3), and our use of an effective dose/selective *β*_1_–blocker (without peripheral vascular effects) strongly support a cause‐effect relationship between acute lowering of cardiac pump function and augmented O_2_ extraction in the active leg muscles. Future studies could assess the effects of acute BP lowering per se (via alpha‐adrenergic blockade; see (Mortensen et al. 2017) on individual muscle NIRS responses to more thoroughly test this hypothesis.

### Effects of esmolol on systemic adjustments to exercise

Systemic oxygen uptake (VO_2_) during the final 20 sec of each work intensity was unaltered by esmolol infusion (Table 2). This finding is consistent with most previous *β*_1_‐blockade studies of healthy subjects during steady state, submaximal exercise (Epstein et al. 1965; Pawelczyk et al. 1992; Tesch 1985) and suggests that gross exercise efficiency is unaltered by acute administration of these cardioselective drugs.

As expected, esmolol reduced the tachycardia of exercise (Fig. 2). We did not directly measure cardiac output for calculations of stroke volume, but did observe consistent and significant increases in O_2_ pulse during the esmolol trial (average of 1.5 to 2.0 mL/beat higher vs. PRE; Table 2). This effect disappeared 45 min after termination of the esmolol maintenance infusion. These O_2_ pulse responses, while indirect (Crisafulli et al. 2007), are consistent with multiple previous investigations that reported increases in stroke volume during light and moderately intense exercise under *β*‐blockade (Tesch 1985).

The significant reduction in systolic BP at both work intensities (Fig. 2) is also comparable to prior studies (Deegan and Wood 1994; Reilly et al. 1985) likely reflecting the negative inotropic effects of esmolol on the heart (Gorcsan et al. 1997; Weidemann et al. 2002). Mean BP was also lower at both workloads during esmolol (data not shown). This reduction in MAP, coupled with the reductions seen in SmO_2_, is consistent with an acute reduction in active muscle blood flow due to esmolol.

### Experimental considerations

As stated in the Methods, subjects in this study were part of a larger investigation examining the HR lowering effects of esmolol versus propranolol (Muller et al. 2017). This dictated the use of absolute HR rather than %VO_2_ peak to establish the two cycle ergometer work rates used in the present analysis. The age range for this larger study was also intentionally broad (22–67 year) and included both sexes, with no control for the menstrual cycle timing in the two younger women. Finally, maximal HR and VO_2_ were determined during treadmill screening testing, and not during recumbent cycle exercise. In combination, these factors created a moderately heterogeneous sample (young men and women in their 20s, and three men in their 60s) and precluded our ability to match relative work intensities (i.e., % of recumbent cycle VO_2_ peak) across subjects. Despite this heterogeneity we observed rapid and reversible effects of esmolol on exercising muscle SmO_2_ in all 8 subjects (Fig. 3), suggesting a clear impact of this short‐acting *β*_1_‐selective antagonist on exercising muscle O_2_ extraction in healthy humans regardless of age.

The NIRS device used in this study detects relative changes in SmO_2_ with good temporal resolution (McManus et al. 2018), but does not allow for adipose tissue thickness (ATT) corrections of SmO_2_. The influence ATT on relative SmO_2_ changes can be seen in Figure 3, where individuals with higher values (particularly the 2 women) tended to have smaller exercise‐induced reductions in SmO_2_. However, no subject in this study had an ATT value (thigh or calf) that exceeded the maximal NIRS signal penetration depth of this device (~12.5 mm). Thus we believe the SmO_2_ data reported in these subjects are reflective of changes within the underlying muscle and not adipose tissue or skin. This is further suggested by the fact that relative changes in SmO_2_ in this study were influenced by (1) exercise intensity (moderate > light in the VL muscle; (Hug and Dorel 2009)), (2) expected muscle group recruitment pattern (VL > MG; (Hug and Dorel 2009)), and (3) drug appearance and removal from the circulation (ON esmolol > OFF esmolol).

Slower adjustments in O_2_ uptake at exercise onset, an effect thought to result specifically from blockade of *β*_1_ receptors (Hughson and Kowalchuk 1991), could have occurred during esmolol infusion. However, we did not design this study or use a system to measure breath‐by‐breath VO_2_ responses. Due to its short half‐life, however, esmolol would be a potentially powerful experimental intervention to explore VO_2_ kinetics of human exercise, which typically requires multiple trials at a given workload measured on the same day.

## Conclusions and Perspectives

In summary, we found that systemic *β*_1_ receptor blockade with esmolol rapidly and reversibly increases O_2_ extraction in the active limb muscles. These findings in healthy, non‐exercise trained adults demonstrate the marked effects that *β*–blocking drugs can have on O_2_ supply‐demand balance in specific limb muscles during exercise at submaximal intensities encountered during everyday life. Detrimental effects of esmolol on steady state exercise responses (e.g., whole body VO_2_/efficiency) were not observed, but future studies should examine whether muscle O_2_ extraction adjustments at the onset of exercise are slowed by these drugs, both in healthy and clinical populations.

Increases in peripheral O_2_ extraction, particularly within the leg muscles, represent an essential physiological adjustment to increases in whole body O_2_ demand elicited by exercise and other activities of daily living. For patients with limited cardiac function (Hughson and Kowalchuk 1991), and for those prescribed/taking *β* blockers on a regular or short‐term basis, this widening of the arteriovenous O_2_ difference becomes even more important. The present finding that healthy non‐medicated adults across a broad age range exhibit rapid and intensity‐dependent reductions in exercising leg SmO_2_ when *β*_1_ receptors are acutely blocked, suggests that human leg muscles are intrinsically well‐equipped to meet these systemic demands.

## Conflict of Interest

None declared.
